# The risk of revision using tourniquet or not in primary total knee replacement: an observational study from the Swedish Knee Arthroplasty Register

**DOI:** 10.2340/17453674.2026.45363

**Published:** 2026-01-23

**Authors:** Annette W-DAHL, Johan KÄRRHOLM, Perna Ighani ARANI, Ola ROLFSON

**Affiliations:** 1 The Swedish Arthroplasty Register; 2 Department of Clinical Sciences Lund, Division of Orthopedics, Lund University, Lund; 3 Institute of Clinical Sciences, Sahlgrenska Academy, University of Gothenburg, Gothenburg; 4 Faculty of Medicine and Health, School of Medical Sciences, Örebro University, Örebro, Sweden

## Abstract

**Background and purpose:**

The use of a tourniquet in knee replacement surgery is debated. Given the conflicting evidence, we aimed to compare the risk of revision after total knee replacements (TKR) with or without the use of tourniquet.

**Methods:**

In this register based observational study, we included the 5 most common cemented primary TKR models due to osteoarthritis reported to the Swedish Arthroplasty Register 2010–2024 and followed them until December 31, 2024. The first revision for implant loosening was the primary outcome. We estimated the cumulative revision rate (CRR) with 95% confidence interval (CI) using the 1–Kaplan–Meier method. We examined the use of a tourniquet regarding the risk of revision using multiple Cox regression analysis to calculate the hazard ratio (HR) with CI and adjusted for potential confounding factors.

**Results:**

Of the 149,616 TKRs included, 65,570 (44%) were with tourniquet and 84,046 (56%) without tourniquet. The CRR was similar at all time-points for all causes and infection; however, CRR started to increase at 6–7 years for implant loosening with use of a tourniquet. In the Cox regression analysis, the use of a. tourniquet was associated with an increased risk of revision for implant loosening after 5 years (HR 1.56, CI 1.06–2.30). There was no difference in revision for all causes (HR 1.07, CI 0.99–1.15) or infection (HR 1.08, CI 0.97–1.21).

**Conclusion:**

The use of a tourniquet was associated with an increased risk of revision for implant loosening after 5 years, while no association was found for all-cause revision or infection. Our results do not support the use of a tourniquet in TKR as a strategy to reduce the risk of revision, either due to all causes, implant loosening, or infection.

The use of a tourniquet has long been considered standard practice in knee replacement. Creating a bloodless surgical field may help reduce overall blood loss and enhance intraoperative visualization. Additionally, it may facilitate deeper cement penetration into the trabecular bone, potentially improving implant fixation and long-term survival [1].

In both the United Kingdom and the United States, tourniquets are employed in approximately 80–90% of TKR procedures [2,3]. In Norway, the proportion of surgeries performed with a tourniquet declined from 65% in 2019 to 53% in 2023 [4]. In Denmark, usage has remained relatively stable at around 50% in recent years [5]. Sweden, by contrast, has seen a marked reduction, from 93% in 2010 to 22% in 2024, indicating a significant shift in clinical practice [6].

The latest Cochrane review suggests that tourniquet use in knee replacement surgery may be associated with an increased risk of serious adverse events such as deep vein thrombosis and pulmonary embolism and infection, without offering clear benefits in terms of treatment outcomes, functional recovery, or quality of life. Additionally, the review highlights the need for additional research on tourniquet use and implant survival, noting a lack of long-term studies in this area [7].

Results from the National Joint Registry (NJR) and a Dutch single-center study showed no benefits on risk of revision using a tourniquet [2,8]. In contrast, a recent study from the Norwegian Arthroplasty Register (NAR) indicated that tourniquet use was associated with lower risk of all-cause revision and tibial component loosening [9].

Whether or not to use a tourniquet during TKR remains a topic of ongoing debate [10]. Given the conflicting evidence regarding revision risk, our study aims to compare the risk of first-time revision following primary cemented TKR performed with vs without tourniquet use.

## Method

### Study design

This observational study was based on prospectively collected data from the Swedish Arthroplasty Register (SAR).

SAR is considered highly valid, encompassing all public and private hospitals performing knee replacements in Sweden with a completeness rate of 97–98% for primary surgeries and 92–95% for revisions between 2012 and 2023 [6].

The study was in accordance with the STROBE statement.

### Patients

Patient characteristics including age, sex, body mass index (BMI), American Society of Anesthesiologists’ physical status classification (ASA class), and previous surgery on the index knee were obtained from the SAR. Surgical variables were also retrieved, including operating time, use of tourniquet, year of surgery, presence or absence of a patellar button in primary surgeries, and timing of the preoperative dose of prophylactic antibiotics (administered 45–30 minutes before surgery start), in accordance with Swedish recommendations [11]. Data on revision procedures and reasons for revision was also obtained. Patient characteristics, plus surgical and prophylactic variables had a completeness rate of 98–100% [6].

The SAR began recording tourniquet use in 2009, documenting whether a tourniquet was applied at any point during surgery or not used at all. This register-based observational study included TKRs performed between 2010 and 2024 due to osteoarthritis (OA), focusing on the 5 most reported cemented primary TKR models in the SAR (NexGen with metal-backed tibial component [MBT] [Zimmer Biomet, Warsaw, IN, USA], PFC Sigma MBT [DePuy Synthes, Raynham, MA, USA], Triathlon MBT [Stryker, Kalamazoo, MI, USA], Persona Knee [Zimmer Biomet], and Genesis II MBT [Smith+Nephew, Watford UK]). Patients were followed until revision, death, emigration, or until December 31, 2024.

The selected prosthesis models may include various component combinations. Models specifically made for revision or surgeries involving extra-long stems (longer than 5 cm) were excluded. The included prosthesis models constitute 86% of all cemented TKRs during the study period.

### Outcomes

The main outcome was first revision for implant loosening,. Further outcomes were first revision for any reason and infection (both verified and suspected). Other reasons for revision were categorized into instability, patellar problems, polyethylene wear/fracture, wear, fracture, and other. Multiple reasons for revision could be registered; however, one is designated as the main reason, verified through medical records and determined according to a predefined hierarchy [12].

Revision procedures were classified into the following categories: exchange of tibial component, exchange of femoral component, exchange of polyethylene insert, exchange to TKR without patella, TKR with patella, stabilized prosthesis, patella addition, extraction of the prosthesis/arthrodesis/amputation, and other.

### Statistics

Patient characteristics, surgical variables, cause of revision, and revision procedure are presented as number with percentage, mean, and standard deviation (SD) or median with interquartile range (IQR), as appropriate. Comparisons of patient characteristics and surgical variables between procedures performed with and without a tourniquet were conducted using standardized mean differences (SMD). We estimated the cumulative revision rate (CRR) with 95% confidence interval (CI) using the 1–Kaplan-Meier method with the endpoints revision for any reason, implant loosening, and infection. We further examined the use of a tourniquet, yes or no, regarding the risk of revision for any reason, using multiple Cox regression analysis to calculate the hazard ratio (HR). We adjusted for potential confounders, age group (< 45, 45–54, 55–64, 65–74, 75–84, and ≥ 85), sex, BMI category (18.5–24.9, 25–29.9, 30–34.9, 35–39.9, and ≥ 40), year of surgery (2010–2017 and 2018–2024), presence of patellar button or not, operating time per 10 minutes and prophylactic antibiotics according to the Swedish recommendations (yes/no). Corresponding Cox regression analyses were performed for revision due to implant loosening and infection. The proportional hazards assumption was assessed and confirmed using residual analysis.

Given the potential interaction between BMI and ASA classification, a sensitivity analysis was performed in which ASA class (I–II and ≥ III) was included instead of BMI category.

Considering missing values of variables included in the Cox regression analyses, BMI was missing in 0.3% of the surgeries, prophylactic antibiotics in 1.3%, and ASA class in 0.2%. The missing values were assumed to be missing at random. We assigned missing values as best scenario (BMI 18.5–24.9, prophylactic antibiotics according to the recommendations as yes, and ASA class I–II). For worst scenario we assigned BMI as > 40 prophylactic antibiotics according to the recommendations as no, and ASA-class ≥ III.

### Ethics, registration, data sharing plan, funding, and potential conflicts of interest

The study was approved by the Swedish Ethical Review Authority (2025-03401-01) and performed according to the Helsinki Declaration. The National Quality Registers are regulated by the Patient Data Act (SFS 2008:355), which does not require written consent from each registered patient. However, patients must be informed that they will be included in the register and that they have the right to opt out. All register-based research with individual data requires approval by the Ethics Review Authority. Data sharing is not possible as the information is secrecy-protected according to the Public Access to Information and Secrecy Act. Microsoft Copilot was used for copyediting the manuscript before submission. The study was in part financed by grants from the Swedish state under the agreement between the Swedish government and the county councils, the ALF agreement (ALFGBG-1006713 and ALFGBG-965217). The authors declare no conflicts of interest. Complete disclosure of interest forms according to ICMJE are available on the article page, doi: 10.2340/17453674.2026.45363

## Results

**Figure 1:**
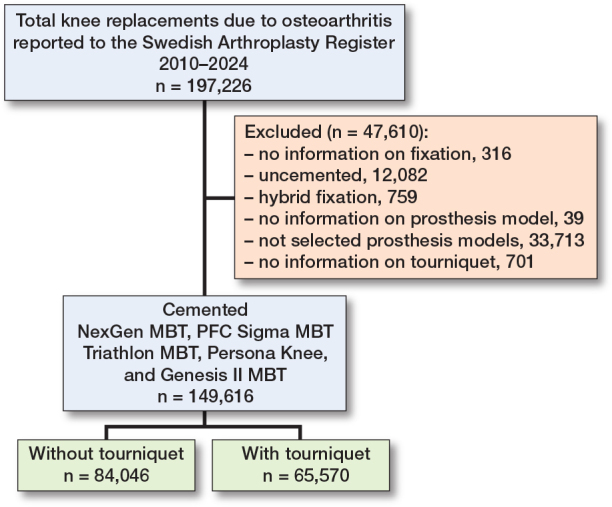
Flowchart of the study. MBT = metal-backed tibial component.

Of the 197,226 TKRs due to OA reported to the SAR 2010–2024, we excluded those with missing data on fixation (n = 316) or prosthesis model (n = 39), uncemented TKRs (n = 12,082), hybrids (n = 759), non-selected prosthesis models (n = 33,713), and missing data on the use of tourniquet (n = 701). Of the 149,616 cemented primary TKRs included, 65,570 (44%) had been inserted with use of a tourniquet and 84,046 (56%) without tourniquet (Figure 1).

**Figure 2:**
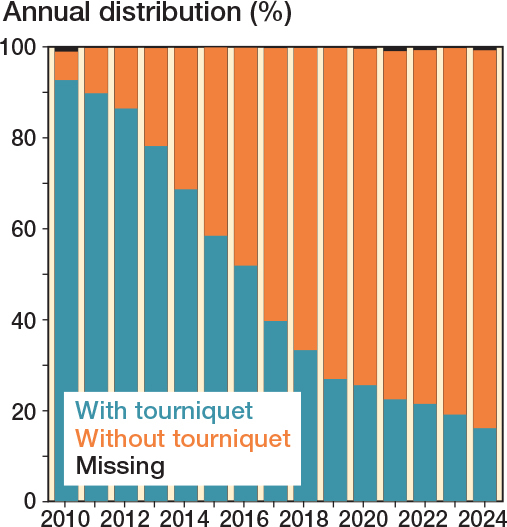
Annual proportion of the use of tourniquet in primary total knee replacement 2010–2024.

The mean age was 70 (SD 8.7) and 57% were female. Patient characteristics and surgical variables were largely similar between the 2 groups, except for operating time and prosthesis model, which showed small and medium effect sizes respectively, based on SMD (Table 1). The use of a tourniquet in TKR declined from 93% in 2010 to 16% in 2024 (Figure 2).

**Table 1:** Patient characteristics and surgical variables in cemented total knee replacements for osteoarthritis 2010–2024 with and without the use of a tourniquet. Values are count (%) or as specified

Factor	Tourniquet	No tourniquet	SMD
Number of procedures	65,570	84,046	
Mean age (SD)	70 (8.6)	70 (8.7)	0.02
Females	37,288 (57)	47,713 (57)	0.002
Mean BMI (SD)	29 (4.5)	29 (4.4)	0.06
ASA-class ≥ III	11,036 (17)	14,744 (18)	0.02
Previous surgery	10,963 (17)	14,290 (17)	0.01
Patellar resurfacing	1,675 (2.6)	1,594 (1.9)	0.05
Operating time minutes,			
median (IQR)	75 (62–93)	68 (54–85)	0.2
Prophylactic antibiotics ^**a**^	31,089 (48)	41,146 (50)	0.03
Prosthesis model			0.5
NexGen MBT	39,925 (61)	50,940 (61)	
PFC Sigma MBT	1,838 (2.8)	6,020 (7.2)	
Triathlon MBT	18,042 (28)	11,347 (14)	
Persona Knee	1,002 (1.5)	2,478 (2.9)	
Genesis II MBT	4,763 (7.3)	13,261 (16)	

a according to recommendations.

ASA = American Society of Anesthesiologists physical status classification, BMI = body mass index, IQR = interquartile range, MBT = metal-backed tibial component. SD = standard deviation, SMD = standardized mean difference,

Nearly 4,000 revisions were reported. The mean follow-up time was longer for those with a tourniquet (9 years) compared with those without a tourniquet (4.7 years). Infection was the most common cause of revision overall, while implant loosening was more common in the group with a tourniquet (Table 2). The most frequent revision procedure in both groups was exchange of the tibial polyethylene insert, followed by TKR exchange without patella and patella addition. Differences in cause of revision and revision procedure were minor (Table 2).

**Table 2:** Cause and procedure of revisions with and without tourniquet. Values are count (%) or as specified

Factor	Tourniquet	No tourniquet	SMD
Number of revisions	2,103	1,831	
Mean follow-up (SD)	9.0 (4.1)	4.7 (3.4)	
Cause			0.2
Infection	837 (40)	810 (44)	
Implant loosening	419 (20)	256 (14)	
Patella	349 (17)	306 (17)	
Instability	345 (17)	325 (18)	
Joint stiffness	35 (1.7)	23 (1.3)	
Fracture	21 (1.0)	32 (1.8)	
Polyethylene wear/fracture	5 (0.2)	6 (0.3)	
Wear	4 (0.2)	0 (0.0)	
Other	79 (3.8)	69 (3.8)	
Revision procedure, n (%)			0.1
Exchange of polyethylene	836 (40)	822 (45)	
TKR exchange without patella	413 (20)	296 (16)	
Patella addition	381 (18)	342 (19)	
Extraction of prosthesis/			
arthrodesis/amputation	162 (7.7)	120 (6.6)	
Exchange of tibial component	93 (4.4)	63 (3.4)	
Stabilized (hinged) prosthesis	97 (4.6)	96 (5.2)	
TKR exchange with patella	90 (4.3)	65 (3.5)	
Exchange of femoral component	23 (1.1)	21 (1.1)	
Other	8 (0.4)	6 (0.3)	

For abbreviations, see Table 1.

### Outcomes

**Figure 3:**
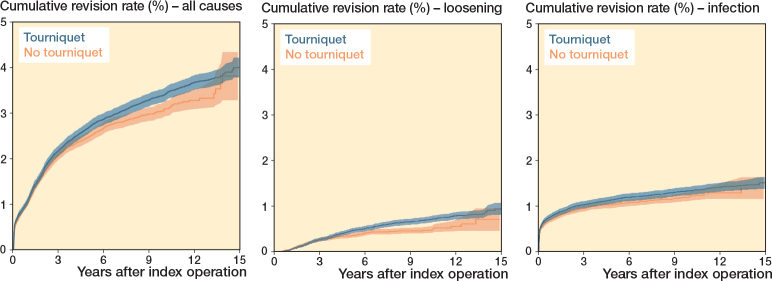
Cumulative revision rate (1–Kaplan–Meier estimate) with 95% confidence interval (until at least 50 at risk) for all causes, implant loosening, and infection for total knee replacements 2010–2024.

CRR for infection was similar across all time points. However, CRR for all causes began to increase more after approximately 9 years, and for implant loosening after 6–7 years in the tourniquet group. At 10 years the CRR for all causes was 3.40 (CI 3.26–3.55) with tourniquet and 3.09 (CI 2.93–3.26) without. For implant loosening, the CRR at 10 years was 0.71 (CI 0.64–0.78) with tourniquet and 0.48 (CI 0.42–0.55) without (Figure 3, Table 3).

**Table 3:** Cumulative revision rate (CRR) and 95% confidence interval at 2, 5, and 10 years for all causes, implant loosening, and infection with and without a tourniquet

CRR at	Tourniquet	No tourniquet
2 years		
All causes	1.74 (1.64–1.84)	1.68 (1.59–1.77)
Implant loosening	0.16 (0.13–0.19)	0.18 (0.15–0.21)
Infection	0.94 (0.87–1.02)	0.87 (0.80–0.93)
5 years		
All causes	2.70 (2.57–2.82)	2.49 (2.37–2.61)
Implant loosening	0.46 (0.41–0.52)	0.37 (0.32–0.42)
Infection	1.14 (1.05–1.22)	1.03 (0.96–1.10)
10 years		
All causes	3.40 (3.26–3.55)	3.09 (2.93–3.26)
Implant loosening	0.71 (0.64–0.78)	0.49 (0.42–0.55)
Infection	1.33 (1.23–1.42)	1.20 (1.10–1.30)

In the Cox regression analyses, adjusted for potential confounders, tourniquet use was associated with no statistical difference in risk of revision for all causes (HR 1.07, CI 0.99–1.15), implant loosening (HR 1.16, CI 0.96–1.39), and infection (HR 1.08, CI 0.97–1.21) (Table 4). Among the confounding factors, the absence of a patellar button at primary surgery was associated with reduced risk across all main outcomes. Increasing age was associated with lower risk for all causes and implant loosening, while surgery performed during 2018–2024 was associated with lower risk for implant loosening. Longer operating time was associated with higher risk for all outcomes. Higher BMI was linked to increased risk for all causes and infection, and female sex was associated with increased risk for implant loosening. After reviewing the Kaplan–Meier curves and completing the planned analyses, we observed a rise in revision rates around 6–7 years postoperatively in the tourniquet group. This prompted a sensitivity analysis with separate Cox regressions for implant loosening before 5 years, after 5 years as well as between 5 and 10 years. The results showed a statistically significant increased risk of revision after 5 years (HR 1.56, CI 1.06–2.30) with tourniquet use, while the risk before 5 years and between 5 and 10 years was elevated (HR 1.19, CI 0.99–1.42 and HR 1.23, CI 0.77–1.97 respectively) but not statistically significant (Table 4). Thus, the difference did not become statistically significant until the follow-up extended past 10 years in our material. A supplementary Cox regression analysis replacing BMI category with ASA class showed marginal changes in results (see Appendix).

**Table 4:** Adjusted hazard ratio (HR) of revision with 95% confidence (CI) interval using tourniquet with no tourniquet as reference for all causes, implant loosening, and infection

Factor	Adjusted ^a^ HR (CI)
All causes	1.05 (0.97–1.12)
Implant loosening	1.17 (0.98–1.38)
Infection	1.08 (0.97–1.21)
< 5 years	1.07 (0.88–1.30)
5–10 years	1.23 (0.77–1.97)
> 5 years	1.56 (1.06–2.30)

a Adjusted for age group, sex, BMI category, surgical year (2010–2017 and 2018–2024), patella at primary surgery (yes/no), operating time per 10 minutes, timing of prophylactic antibiotics according to the Swedish recommendations of 45–30 minutes (yes/no).

Missing values were imputed using both best-case (BMI 18.5–24.9, prophylactic antibiotics administered according to recommendations, ASA class I–II) and worst-case scenarios (BMI > 40, prophylactic antibiotics not administered according to recommendation, ASA class ≥ III). The adjusted hazard ratios varied by no more than one-hundredth (see Appendix).

## Discussion

This is the largest study on tourniquet use in TKR with relatively long follow-up: 9 years for the tourniquet group and 4.7 years for the non-tourniquet group.

With the aim of analyzing the risk of revision in primary TKR due to OA with or without the use of a tourniquet, we found an increased risk of revision due to implant loosening, observed after 5 years in procedures performed with a tourniquet, but similar risk of all-cause revision and for infection.

### Implant loosening

The use of a tourniquet is commonly advocated to improve the cementation quality and thereby the fixation of cemented prostheses and improve the long-term implant survival [1]. However, several studies using radiostereometric analysis (RSA) have failed to demonstrate improved tibial component fixation with tourniquet use [13-15]. The Enhanced Recovery After Surgery (ERAS) Society recommends against routine use of a tourniquet, based on moderate evidence, with a strong recommendation grade [16].

A recent study from the Norwegian Arthroplasty Register (NAR), including 24,000 cemented and hybrid TKRs performed between 2019 and 2023, found that tourniquet use was associated with a reduced risk of tibial loosening. However, the follow-up was relatively short (median 1.9–2.3 years), and 62% of procedures were performed with a tourniquet [9]. In contrast, our study found an increased risk of revision due to implant loosening after 5 years in the tourniquet group.

### All-causes revision

When analyzing all-cause revision, the NAR study reported a reduced risk with tourniquet use, while our findings showed no statistically significant difference between groups. Supporting our results, a study from the National Joint Registry (NJR) found no association between tourniquet use and revision risk. This study included approximately 17,000 cemented TKRs performed in 2003, of which 95% were conducted with a tourniquet, and had a median follow-up of 12 years [2]. Similarly, a single-center study from the Netherlands found no association between tourniquet use and revision risk at 5 years, based on a cohort of just over 500 TKRs [8].

The discrepancies between the Norwegian and Swedish findings may be due to several factors. The number of revisions in the NAR study was relatively low, particularly for implant loosening. Furthermore, the NAR study included all cemented and hybrid TKRs and all diagnoses, while our study focused on the most used and well-documented cemented TKRs for OA. Although the Norwegian study adjusted for multiple variables, the influence of rarely used, lower-performing implants cannot be excluded.

### Infection

Neither our study nor the Norwegian study found an association between tourniquet use and the risk of revision due to infection. According to the meta-analysis of RCTs by Magan et al. (2022), evidence regarding infection risk with tourniquet use is limited. None of the included RCTs had infection as the primary outcome and follow-up times were insufficient. The latest Cochrane review reported a significantly higher risk of wound infection with tourniquet use compared with procedures without, although data on deep infections was lacking [17].

Timing of preoperative prophylactic antibiotics is critical to ensure adequate tissue concentration before tourniquet application or surgery start and depends on the antibiotic’s half-life. In Sweden, cloxacillin (short half-life, ~30 minutes) is recommended, with the first dose administered 45–30 minutes prior to tourniquet [6]. In Norway, compliance is unknown, but first-generation cephalosporins with longer half-lives are commonly used, and recommended to be administered more than 30 minutes before tourniquet application or surgery.

### Other complications

The use of a tourniquet significantly reduced bleeding, postoperative hemoglobin loss, and improved quadriceps strength at 8 weeks,, and showed similar postoperative pain, range of motion, legth of stay, and thromboembolic risk as without the use of a tourniquet [18,19]. Reported disadvantages of tourniquet use include increased risk of complications, greater postoperative pain, reduced range of motion, and longer hospital stays [1,8]. These variables cannot be evaluated using SAR data, except for postoperative pain, which is assessed 1 year postoperatively and may not accurately reflect tourniquet use. Additionally, patient-reported outcome measures (PROMs) were not collected from all units before 2021, and overall completeness is approximately 65% [6].

### Use of a tourniquet

Surgeons may choose to use a tourniquet based on training, habit, preference for a bloodless field, or perceived reduction in operating time. In Sweden, tourniquet use in TKR has declined, from 93% in 2010 to 16% in 2024. The shift in the use of a tourniquet as well as changes in surgical technique and implant over the years must be considered when the evaluation extends over a long period. This was addressed by adjusting for surgical year as a time-dependent covariate in the Cox regression analysis. In 2024, half of the surgical units (39/80) reported no use of a tourniquet, compared with none in 2010. Additionally, 24/80 units (30%) reported tourniquet use in 1–10 surgeries, and 17/80 units (21%) in 50–100%. Units with high tourniquet use in 2024 had previously reported 83–100% usage in 2010. This decline likely reflects a shift in practice among Swedish knee surgeons, as the rationale for tourniquet use is increasingly unsupported by the literature and associated with adverse events. Improvements in surgical technique, anesthesia, and the use of tranexamic acid may also have contributed to this change.

### Strengths

The main strengths are high coverage and completeness for both primary and revision procedures, a high response rate regarding tourniquet use, and verification of revision causes via medical records. The 5 selected cemented prosthesis models are well documented and accounted for 86% of cemented TKRs reported to the SAR during the study period. The 10-year CRR for all cemented TKRs between 2014 and 2024 was estimated at 3.3% (CI 3.1–3.4), consistent with our findings: 3.4% (CI 3.25–3.55) with tourniquet and 3.09% (CI 2.93–3.26) without.

### Limitations

Our analysis is subject to confounding by indication and residual confounding. We lacked information on whether the tourniquet was used throughout the entire procedure or only partially. Missing data was low (< 2%) and addressed by conducting best- and worst-case scenario analyses, which did not alter the results. Furthermore, we had no data on the use of tranexamic acid or complications potentially related to tourniquet use, such as deep vein thrombosis or pulmonary embolism.

### Conclusion

The use of a tourniquet was associated with an increased risk of revision due to implant loosening after 5 years, while no association was found for all-cause revision or infection. Based on these findings, tourniquet use in TKR seems not supported as a strategy to reduce the risk of revision—either overall, or for implant loosening or infection.

## Notes

The study was designed by AWD and OR. AWD, OR, JK, and PIA prepared the analysis plan. AWD, OR, and JK analyzed the data. AWD prepared the manuscript. AWD, OR, JK, and PIA participated in data interpretation, critically revised the manuscript, and approved the final version.

The authors thank the contact secretaries and contact orthopedic surgeons around Sweden for their excellent work and commitment to the SAR. Thanks are also offered to Alexander Thorén for support with the statistical analyses.

Handling co-editors: Per Henrik Randsborg

*Acta* thanks Sarunas Tarasevicius and other anonymous reviewers for help with peer review of this manuscript.

## Appendix

**Table:** Adjusted hazard ratio (HR) of revision with 95% confidence (CI) interval using tourniquet with no tourniquet as reference for all causes, implant loosening, and infection after including ASA class instead of BMI and as best-case and worst-case scenario respectively

	ASA class instead of BMI	Best-case scenario	Worst-case scenario
Factor	Adjusted ^a^ HR (CI)	Adjusted ^a^ HR (CI)	Adjusted ^a^ HR (CI)
All causes	1.06 (0.98–1.13)	1.05 (0.98–1.13)	1.05 (0.98–1.18)
Implant loosening	1.18 (0.99–1.40)	1.16 (0.98–1.38)	1.16 (0.98–1.38)
Infection	1.10 (0.99–1.23)	1.09 (0.98–1.21)	1.09 (0.98–1.21)

For abbreviations, see Table 4.
